# Efficient Protein Expression and Virus-Induced Gene Silencing in Plants Using a Crinivirus-Derived Vector

**DOI:** 10.3390/v10050216

**Published:** 2018-04-24

**Authors:** Wenjie Qiao, Bryce W. Falk

**Affiliations:** Department of Plant Pathology, University of California, Davis, 95616 CA, USA; qwenjie@ucdavis.edu

**Keywords:** *Crinivirus*, *Lettuce infectious yellows virus*, protein expression, virus-induced gene silencing

## Abstract

Plant virus-based vectors are valuable tools for recombinant gene expression and functional genomics for both basic and applied research. In this study, *Lettuce infectious yellows virus* (LIYV) of the genus *Crinivirus* was engineered into a virus vector that is applicable for efficient protein expression and virus-induced gene silencing (VIGS) in plants. We examined gene replacement and “add a gene” strategies to develop LIYV-derived vectors for transient expression of the green fluorescent protein (GFP) reporter in *Nicotiana benthamiana* plants. The latter yielded higher GFP expression and was further examined by testing the effects of heterologous controller elements (CEs). A series of five vector constructs with progressively extended LIYV CP sgRNA CEs were tested, the longest CE gave the highest GFP expression but lower virus accumulation. The whitefly transmissibility of the optimized vector construct to other host plants, and the capability to accommodate and express a larger gene, a 1.8 kb β-glucuronidase (GUS) gene, were confirmed. Furthermore, the LIYV vector was also validated VIGS by silencing the endogenous gene, *phytoene desaturase* (PDS) in *N. benthamiana* plants, and the transgene GFP in *N. benthamiana* line 16c plants. Therefore, LIYV-derived vectors could provide a technical reference for developing vectors of other economically important criniviruses.

## 1. Introduction

Plant virus-based vectors are important tools for gene expression and silencing in plants, and have been widely applied for both fundamental and applied research including tracking virus movement and distribution in plants [1,2,3], examining gene/protein functions by over-expression or silencing [4,5,6,7], triggering RNA interference (RNAi)-mediated plant protection against pathogens or insects [8,9,10,11,12], and producing commercial products such as enzymes, immunogens or antibodies [13,14,15,16,17]. As a convenient laboratory tool for molecular research and as an alternative to generating transgenic plants for various purposes, many RNA and DNA plant viruses with different host ranges have been engineered and the list is still growing [3,18,19,20].

Criniviruses, the whitefly-transmitted members in the family *Closteroviridae*, cause phloem-limited infections in various important crops worldwide such as lettuce, tomato, cucurbits, and sweet potato, that lead to severe diseases and significant economic losses [21]. Viruses in the genus *Crinivirus* are characterized by their large bipartite genome of positive-sense single-stranded RNA totaling approximately 15.3–17.7 kb. The overall genomic organization is similar for all viruses in the genus, *Lettuce infectious yellows virus* (LIYV) is the type member [22]. LIYV RNA1 is 8118 nt and encodes proteins associated with replication and alone is competent for replication: ORFs 1a and 1b code for the conserved domains of papain-like cysteine proteinase (PRO), methyltransferase (MTR), helicase (HEL) and RNA-dependent RNA polymerase (RdRp), P34 encoded by ORF2 is translated from its subgenomic RNA (sgRNA) and is required for LIYV RNA2 replication [23,24]. RNA2 is 7193 nt and contains seven ORFs encoding proteins relevant to virion encapsidation, movement and vector transmission: the CP (major coat protein), CPm (minor coat protein), Hsp70h, and P59 are virion structural components, only CPm can be partially deleted and retain systemic infection in plants but shows disrupted whitefly transmissibility [25,26]; P26 induces plasmalemma deposit and is required for systemic plant infection [27]; two remaining small proteins, P5 and P9, are two nonstructural proteins not required for systemic infection, although when deleted, both showed alleviated LIYV symptoms and decreased virus accumulation levels in plants. P5 is likely translated directly from genomic RNA2, while six 3′-coterminal sgRNAs produced from RNA2 serve as mRNAs for the remaining six genes and their expression is regulated by the cis-acting sequences located immediately upstream of their ORFs, referred as ‘controller elements’ (CE) [28,29].

So far, for viruses of the *Closteroviridae*, only three viruses, all of the genus *Closterovirus*, have been developed as virus vectors. These have proved to be useful for fundamental and translational studies, but all have relatively limited plant host ranges. By contrast, LIYV has a relatively wide host range among herbaceous plant species, and unlike viruses of the genus *Closterovirus*, criniviruses such as LIYV have 2 genomic RNAs and shorter virions, thus offering different opportunities as a virus-based expression vector. In this work, we examined strategies for constructing a LIYV-based vector for transient reporter expression in plants utilizing the green fluorescent protein (GFP) and β-glucuronidase (GUS) genes, thus facilitating gene expression quantification and the monitoring the virus infection progress. We also assessed whether the LIYV-based vector might be useful as a tool for virus induced gene silencing (VIGS) to knock-down target gene expression through RNA interference (RNAi) for conveying gene functions [30,31,32]. Here, we showed that LIYV can be used both for recombinant protein expression and as a VIGS vector, and that the recombinant virus retains whitefly vector transmissibility allowing for testing in various plant species.

## 2. Materials and Methods

### 2.1. Plant Materials

The HC-Pro transgenic *Nicotiana benthamiana* plants overexpressing the silencing suppressor P1/HC-Pro of *Turnip mosaic virus* were commonly used in this study to enhance LIYV accumulation and symptom development [10]. The 16c transgenic *N. benthamiana* plants expressing GFP were used to test the effects of LIYV-VIGS construct [33]. Seeds from both transgenic plants were germinated on KCMS medium containing kanamycin antibiotic (50 mg/mL), after transplanting onto soil, they were kept in growth chamber with 24 °C constant temperature, 60% humidity and 16/8 h daylight cycle. Lettuce (*Lactuca sativa* L.) and *Chenopodium murale* plants were kept in greenhouse at 20–25 °C with 14 h light period.

### 2.2. LIYV Constructs

**Figure 1:**
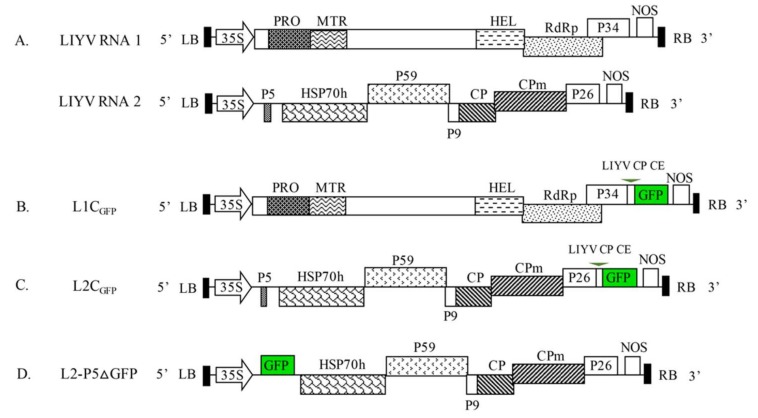
Schematic diagram of the genome organization of lettuce infectious yellows virus (LIYV) cDNA infectious clones (**A**), and their derivatives encoding green fluorescent protein (GFP): (**B**) L1C_GFP_, the GFP ORF under a duplicated LIYV CP subgenomic RNA controller element (LIYV CP CE) inserted between P34 ORF and 3′-nontranslated region (NTR) of LIYV RNA1; (**C**) L2C_GFP_, the GFP ORF under a duplicated LIYV CP CE inserted between P26 ORF and 3′NTR of LIYV RNA2; (**D**) L2-P5ΔGFP, the P5 gene of LIYV RNA2 replaced by the GFP ORF. LB, left border; RB, right border; 35S, 35S promoter; NOS, nopaline synthase terminator; the boxes indicate open reading frames (ORF) and their corresponding translation products: PRO, papain-like cysteine proteinase; MTR, methyltransferase; HEL, helicase; POL, RNA-dependent RNA polymerase; HSP70h, heat shock protein 70 homolog; CP, capsid protein; CPm, minor capsid protein; P, proteins named by their approximate molecular mass (e.g., P34, 34-kDa protein).

The full-length cDNA clones of LIYV-WT were the basis of all constructs in this study (Figure 1) [10]. All the constructs were generated through one or two steps using In-Fusion HD Cloning Kit following manufacturer’s instructions (Clontech, Mountain View, CA, USA), and all the plasmid backbones or insertion sequences needed were amplified using CloneAmp HiFi PCR Premix (Clontech). For creating constructs by gene insertion, the ORFs of cycle 3 GFP (a GFP mutant with improved brightness and eliminated tendency to aggregate [34,35]) and GUS, or the truncated *PDS* and *GFP* sequences were first cloned into LIYV RNA1 or RNA2 clones, followed by introducing the CE sequence selected in front of CP genes of LIYV (Accession # U15441, 4071–4220 nt), BYV (Accession # AF190581, 13,547–13,641 nt; [36]) and CYSDV (Accession # FJ492808, 4777–4926 nt). To generate the P5 substitution construct, the GFP ORF was simply ligated into the linearized plasmid of LIYV RNA2 clone without the P5 ORF through In-Fusion Cloning. All clones were sequenced to verify the accuracy of insertions prior to further analysis.

### 2.3. LIYV Inoculation

LIYV constructs were delivered to the target plants by agroinoculation or whitefly (*Bemisia tabaci*) transmission. For *N. benthamiana* plants, LIYV constructs were transformed into *Agrobacterium tumefaciens* (strain GV3101) and agroinfiltrated into plants at 4–6 leaf stages as described before [10]. For lettuce and *C. murale* plants, LIYV virions were purified from systemically infected *N. benthamiana* plants following the protocol described by Klaassen, et al. [37]. After a 6 to 10 h acquisition period on an artificial sucrose diet containing LIYV particles, the viruliferous *B. tabaci* were transferred onto six young lettuce plants and six *C. murale* plants for virus inoculation [25]. Whiteflies were eliminated with insecticide after a three-day inoculation access period.

### 2.4. Fluorescence Detection

GFP fluorescence of the whole plants or the intact leaves and flowers was viewed with a long-wavelength UV light and was photographed directly using a Canon EOS 600D digital camera. Images of fluorescence distribution in hand-cut cross and longitudinal sections of petioles and roots from LIYV-L2C_GFP_ infected *N. benthamiana* plants were taken with a Leica MZFIII fluorescence stereomicroscope (Leica Microsystems, Wetzlar, Germany) provided with UV illumination and a GFP filter. To observe the magnified leaf veinlets, the epidermal cells were removed from the lower side of leaf tissue and mounted in water. GFP fluorescence was observed using a Leica DM5000 B fluorescence microscope (Leica Microsystems).

### 2.5. RT-PCR and RT-qPCR

For RT-PCR and real-time RT-qPCR, total RNAs were extracted from upper non-inoculated leaves using the TRIzol reagent (Invitrogen, Carlsbad, CA, USA) and treated with DNase (QIAGEN, Hilden, Germany) to remove contaminating DNA. Systemic infection and foreign gene stability were checked by RT-PCR, the first-strand cDNAs were generated with SuperScript™ II Reverse Transcriptase (Invitrogen), and PCR was carried out to amplify the partial LIYV CP-coding region (530 bp) using the primer set LIYV-CP_F (5′-TTGTCCAAAATCGTTGTACGCG-3′) and LIYV-CP_R (5′-CCACCTTCACCTTTGCATAATG-3′). For all the “add a gene” constructs of LIYV RNA2, the insertions were checked with primers flanking the inserted segment, LIYV-IN_F (5′-CACGATCTCTCCTTTAGATAAGCAAAGG-3′) and LIYV-IN_R (5′-ATCGAATAATTCAATCACCACTCTCTGATCA-3′).

For real-time RT-qPCR to determine the viral RNA accumulation level, cDNA synthesis was performed with iScript reverse transcription supermix (Bio-Rad, Hercules, CA, USA), and real-time qPCR reactions were assembled with iTaq universal probes supermix (Bio-Rad), the relative LIYV RNA accumulation level was determined using RNA1-specific primers LIYV RNA1_F (5′-TGTTCGCCCAGGT-TAGATTTG-3′), LIYV RNA1_R (5′-TTCACCATATCCTTTCAGCCC-3′) and LIYV RNA1_Probe (5′-AGACACATCCAAAGGGCCACAGT-3′), the *N. benthamiana* protein phosphatase 2A (PP2A) transcripts quantified with PP2A_F: 5′-GAGAAAACCATTCGCCCTAGT-3′, PP2A_R: 5′-GACTGAAGTGCTTGATTGGC-3′ and PP2A_Probe: 5′-CTGAAGACCCTGATGTTGATGTTCGCT-3′ was used as an internal control. QPCR was performed and analyzed with a CFX96 real-time PCR detection system (Bio-Rad) following the manufacturer’s recommendations.

### 2.6. Immunoblot Analysis

Immunoblot analysis was employed to confirm successful LIYV infection and GFP accumulation. Total proteins of 0.1 g plant material were extracted in 100 mM Tris-HCl (pH 7.5), 100 mM EDTA (pH 8.0), 5 mM dithiothreitol, 150 mM NaCl, and 0.1% (*v*/*v*) Triton X-100, separated by SDS-PAGE, and transferred to nitrocellulose membranes [38]. LIYV CP and exogenous GFP protein were detected by immunoblotting using LIYV CP and GFP specific antibodies produced in rabbit. Horseradish peroxidase-coupled goat anti-rabbit IgG (Bio-Rad) was used as the secondary antibody. The blots were treated with chemiluminescence detection reagents (Thermo, Waltham, MA, USA) for visualization.

### 2.7. GUS Assays

GUS histochemical staining was performed on *N. benthamiana* leaves and hand-cut sections of petioles and stems using assays modified from Lagarde et al. [39]. In brief, plant tissues were vacuum infiltrated with GUS staining buffer comprising 50 mM sodium phosphate (pH 7.0), 10 mM EDTA (pH 8.0), 0.1% (*v*/*v*) Triton X-100, 0.5 mg/mL X-Gluc. The enzymatic reaction was performed at 37 °C overnight in the dark. Tissue pigments were removed through a graded ethanol series. The GUS activity was viewed and directly photographed.

## 3. Results

### 3.1. Addition of the GFP Gene to the LIYV Genome

**Figure 2:**
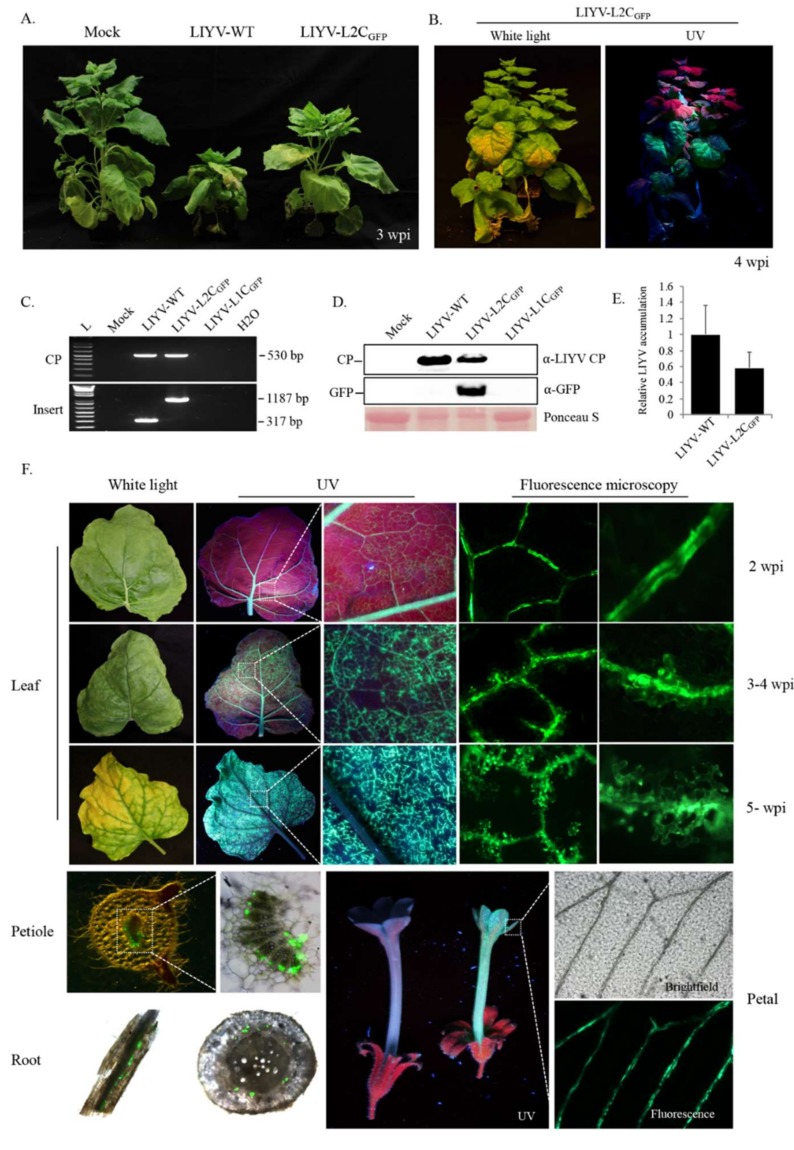
Viral infection and GFP expression of LIYV-based “add-a-gene” expression vectors in Hc-Pro *Nicotiana benthamiana* plants. (**A**) Phenotypes of LIYV-WT and LIYV-L2C_GFP_ infected *N. benthamiana* plants photographed at 3 weeks post inoculation. Mock indicates buffer-inoculated control. (**B**) LIYV-L2C_GFP_ infected *N. benthamiana* plant photographed under white and UV light. (**C**) Detection of viral infection and insertion integrity by RT-PCR with total RNA extracted from upper non-inoculated leaves of LIYV-WT, LIYV-L1C_GFP_, and LIYV-L2C_GFP_ agroinoculated plants. Two primer sets were used to amplify the sequence of LIYV CP (CP, 530 bp) and the sequence flanking the GFP cassette (Insert, 1187 bp), LIYV-WT without the insert was amplified as a control (317 bp). (**D**) Immunoblot analysis of LIYV CP and GFP accumulation in upper non-inoculated leaves using LIYV CP and GFP specific antibodies. The Ponceau S stained rubisco large subunit serves as a loading control. (**E**) Quantification of LIYV RNA1 accumulation in LIYV-WT and LIYV-L2C_GFP_ infected plants by RT-qPCR. The PP2A transcript level of *N. benthamiana* was used as an internal control. Error bars denote standard errors from at least three biological replicates. (**F**) Systemic spread and distribution in leaves, petioles, roots and flowers of LIYV-L2C_GFP_ infected plants monitored by GFP fluorescence. Leaf, progress of LIYV infection and accumulation along veins over time. GFP fluorescence was visualized under UV light and fluorescence microscopy with different magnification. GFP was confined to vascular tissues of petiole, root (left, root segment; right, root cross-section), and petal was seen under UV light and fluorescence microscopy. Dotted lines indicate enlarged areas.

The “add-a-gene” strategy, i.e., engineering of an autonomous expression cassette controlled by an additional sgRNA promoter or CE, is preferred in developing closterovirus-based vectors for stable and high-level expression [40]. Genes placed closer to the 3′ terminus of the closterovirus genomic RNA tend to be expressed in greater amounts [36]. Therefore, for the bipartite LIYV genome, we inserted a GFP expression cassette, including the GFP ORF and a homologous duplication of 150 nt upstream of the LIYV CP ORF as the sgRNA CE, into the LIYV infectious cDNA clones. The cassette was inserted between the P34 ORF and 3′-nontranslated region (NTR) of RNA1, and between the P26 ORF and 3′NTR of RNA2, referred to as L1C_GFP_ and L2C_GFP_ respectively (Figure 1). The combinations of the wild-type RNA1/RNA2 (LIYV-WT), L1C_GFP_/RNA2 (LIYV-L1C_GFP_) and RNA1/L2C_GFP_ (LIYV-L2C_GFP_) were delivered respectively to Hc-Pro transgenic *N. benthamiana* plants by agroinfiltration. At 3 weeks post inoculation (wpi), typical LIYV symptoms were observed in LIYV-L2C_GFP_ inoculated plants, although these appeared milder and about one week later than symptoms caused by LIYV-WT (Figure 2A). The GFP fluorescence was observed in LIYV-L2C_GFP_ infected plants by using a handheld long-wave UV light, the brightest fluorescence was in areas that showed typical LIYV-induced leaf yellowing (Figure 2B). However, no viral symptoms or GFP expression were achieved with LIYV-L1C_GFP_ inoculated plants. Viral infection, insert retention and GFP accumulation from LIYV-L2C_GFP_ in upper leaves were confirmed by RT-PCR with primers amplifying the sequences of LIYV CP and the GFP expression cassette, and by immunoblot analysis using antibodies specific to LIYV CP and GFP (Figure 2C,D). LIYV RNA accumulation was quantified by RT-qPCR, consistent with symptom severity, LIYV RNA titer was almost twice higher in LIYV-WT infected plants than that in LIYV-L2C_GFP_ infected plants (Figure 2E). LIYV-L2C_GFP_ distribution in tissues of systemically infected *N. benthamiana* plants was monitored by fluorescence microscopy. GFP fluorescence initially (2 to 3 wpi) showed in stems and was unevenly distributed limited to the main veins or veinlets of medium and upper leaves. After another 1 to 2 weeks, GFP fluorescence appeared brighter in the leaves but remained phloem-limited. This was revealed by the bright fluorescence observed in leaves, leaf petioles, roots and flowers of *N. benthamiana* plants (Figure 2F).

### 3.2. Substitution of an LIYV ORF with the GFP ORF

**Figure 3:**
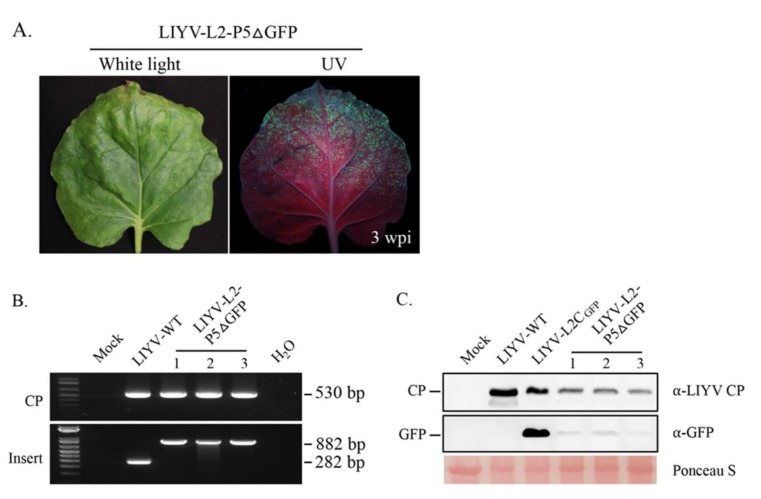
Viral infection and GFP expression of LIYV-based “ORF substitution” expression vector in Hc-Pro *Nicotiana benthamiana* plants. (**A**) LIYV symptoms in upper non-inoculated leaves of LIYV-L2-P5ΔGFP infected plants at 3 wpi and GFP fluorescence visualized under UV light. (**B**) Detection of viral infection and insertion integrity by RT-PCR using total RNA extracted from upper non-inoculated leaves of LIYV-WT and LIYV-L2-P5ΔGFP agroinoculated plants. Two primer sets were used to amplify the sequence of LIYV CP (CP, 530 bp) and the sequence flanking the GFP ORF (Insert, 882 bp), LIYV-WT without the GFP ORF was used as a control (282 bp). (**C**) Immunoblot analysis of LIYV CP and GFP accumulation in upper non-inoculated leaves infected by LIYV-WT, LIYV-L2C_GFP_, and LIYV-L2-P5ΔGFP using LIYV CP and GFP specific antibodies. The Ponceau S stained rubisco large subunit serves as a loading control.

Another common way to construct viral vectors for foreign protein expression is to substitute a viral gene that is not necessary for replication and movement with the gene for the desired protein. LIYV only has two genes encoded by RNA2, P5 and P9, that can be eliminated without disrupting systemic infection in *N. benthamiana* plants, but these mutations lead to decreased symptoms and virus accumulation. P5 is encoded by the first ORF of LIYV RNA2, while P9 ORF is positioned near the middle of RNA 2 with its 5′-terminus overlapping the P59 gene and its 3′-terminus 3 nt away from CP ORF. Considering the complexity of the location of P9 ORF, we chose to replace the P5 ORF with the GFP ORF, resulting in construct L2-P5ΔGFP. When *A. tumefaciens* harboring L2-P5ΔGFP was co-infiltrated with the wild-type RNA1 into Hc-Pro *N. benthamiana* plants, GFP expression was observed in upper non-inoculated leaves under UV light along with the development of LIYV symptoms, however, the fluorescence was much weaker than that of LIYV-L2C_GFP_ at the same infection stage (Figure 3A). The systemic infection of LIYV-L2-P5ΔGFP was confirmed by RT-PCR with total RNA isolated from upper leaves using LIYV CP primers, while the presence of the GFP cassette was determined using primers flanking the original P5 ORF. The expected PCR products of 282 bp for LIYV-WT and 882 bp for LIYV-L2-P5ΔGFP were detected (Figure 3B). LIYV CP and GFP accumulation levels in LIYV-L2-P5ΔGFP infected plants were further determined by immunoblot analysis, both were much lower compared to those in plants infected by LIYV-WT and LIYV-L2C_GFP_ (Figure 3C). These results demonstrated that GFP expression can be achieved by substituting LIYV P5 ORF with GFP ORF but at a lower efficiency comparing with the “add-a-gene” construct LIYV-L2C_GFP_.

### 3.3. Effects of Heterologous CP CEs

**Figure 4:**
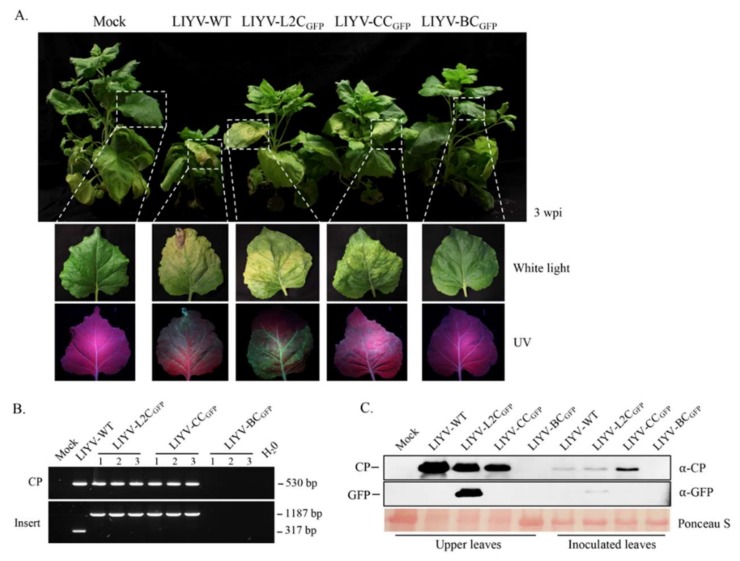
Effects of heterologous CP subgenomic controller elements (CEs) on LIYV infection and GFP expression in Hc-Pro *Nicotiana benthamiana* plants. (**A**) Phenotypes of *N. benthamiana* plants inoculated with buffer (Mock), LIYV-WT, LIYV-L2C_GFP_ (LIYV CP CE), LIYV-CC_GFP_ (CYSDV CP CE), and LIYV-BC_GFP_ (BYV CP CE) at 3 wpi. Symptoms on upper leaves (white light) and GFP fluorescence visualized under UV light (lower panels). Dotted lines indicate enlarged areas. (**B**) Detection of viral infection and insertion integrity by RT-PCR with total RNA extracted from upper non-inoculated leaves. Two primer sets were used to amplify the sequence of LIYV CP (CP, 530 bp) and the sequence flanking the GFP cassette (Insert, 1187 bp), LIYV-WT without the insert was amplified as a control (317 bp). (**C**) Immunoblot analysis of LIYV CP and GFP accumulation with total protein extracts from agroinoculated and upper non-inoculated leaves using LIYV CP and GFP specific antibodies. The Ponceau S stained rubisco large subunit serves as a loading control.

Duplication of the sgRNA CE has been found to cause homologous recombination resulting in the loss of the inserted sequences, while the use of a heterologous sgRNA CE from related viruses were relatively stable [41]. To determine whether this was also the case for LIYV, the homologous duplication of the 150 nt LIYV CP sgRNA CE in L2C_GFP_ was replaced by the heterologous CP sgRNA CE of *Beet yellows virus* (BYV, genus *Closterovirus*) and *Cucurbit yellowing stunting disorder virus* (CYSDV, genus *Crinivirus*), assembled as BC_GFP_ and CC_GFP._ *A. tumefaciens* cells harboring these constructs together with the wild-type LIYV RNA1 clone were infiltrated into leaves of Hc-Pro *N. benthamiana* plants and symptom development and GFP expression were monitored over time. LIYV symptoms were observed on LIYV-WT, LIYV-L2C_GFP_ and LIYV-CC_GFP_ inoculated plants, whereas no symptoms of infection were seen for the LIYV-BC_GFP_ construct (Figure 4A). Surprisingly, the GFP fluorescence was only detected for LIYV-L2C_GFP_ infected plants under the UV light (Figure 4A). Systemic infection and protein expression were confirmed by RT-PCR and immunoblot analysis. Consistent with the symptom development, the sequences of LIYV CP and the inserted cassette were detected within upper non-inoculated leaves of LIYV-WT, LIYV-L2C_GFP_ and LIYV-CC_GFP_ infected plants, indicating systemic infection of these constructs and integrity of the insertions, and no LIYV RNA was detected from LIYV-BC_GFP_ inoculated plants (Figure 4B). Western blot analysis performed with total protein extracts from agroinoculated and upper non-inoculated leaves showed that both LIYV CP and GFP were detected in LIYV-L2C_GFP_ infected leaf samples, and although a similar CP expression level was found in LIYV-CC_GFP_ infected plants, no GFP expression was detected (Figure 4C). As expected, no LIYV infection was detected with LIYV-BC_GFP_ inoculated plants.

### 3.4. Effects of the Size of LIYV CP CE

**Figure 5:**
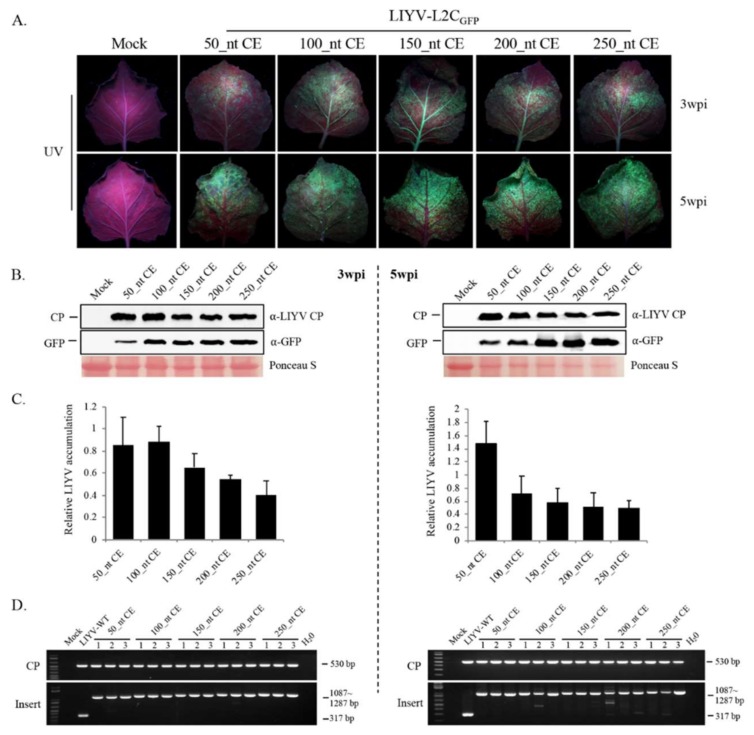
Effects of the size of LIYV CP subgenomic controller element (CE) on viral infection and GFP expression in Hc-Pro *Nicotiana benthamiana* plants. (**A**) GFP fluorescence visualized under UV light on upper leaves of *N. benthamiana* plants infected by five LIYV-L2C_GFP_ constructs comprising CP CEs ranging from 50–250 nt at 3 wpi and 5 wpi. (**B**) Immunoblot analysis of LIYV CP and GFP accumulation with total protein extracts from upper non-inoculated leaves at 3 wpi and 5 wpi using LIYV CP and GFP specific antibodies. The Ponceau S stained Rubisco large subunit serves as a loading control. **(C)** Quantification of LIYV RNA1 accumulation with total RNA isolated from upper non-inoculated leaves at 3 wpi and 5 wpi through RT-qPCR. The PP2A transcript level of *N. benthamiana* was used as an internal control. Error bars denote standard errors from at least three biological replicates. (**D**) Detection of viral infection and insertion integrity by RT-PCR with total RNA extracted from upper non-inoculated leaves at 3 wpi and 5 wpi. Two primer sets were used to amplify the sequence of LIYV CP (CP, 530 bp) and the sequence flanking the GFP cassette (Insert, ranging from 1087–1287 bp), LIYV-WT without the insert was amplified as a control (317 bp).

The 150 nt CE sequence for LIYV-L2C_GFP_ was first selected as an arbitrary sequence upstream of the LIYV CP ORF. To examine the effects of different lengths of this upstream sequence, another four constructs ranging from 50–250 nt inserted in front of the GFP ORF were tested. All five gave systemic infections and GFP expression in agroinoculated Hc-Pro N. benthamiana plants at 2 to 3 wpi. The shortest 50 nt CE insertion construct showed the most severe symptoms but the weakest GFP fluorescence, and decreasing symptom severity and increasing GFP fluorescence were roughly correlated with increasing CE length (Figure 5A). LIYV CP and GFP accumulation were analyzed in systemically infected leaf tissues at 3 wpi and 5 wpi by immunoblot analysis with anti-LIYV CP and anti-GFP antibodies. The 50 nt and 100 nt CE constructs showed high expression of LIYV CP, but reductions of GFP expression, especially at 5 wpi. By contrast, the 200 nt and 250 nt CP CE constructs showed similar CP and GFP accumulation comparable to that of 150 nt CE (Figure 5B). LIYV quantification for these five constructs by RT-qPCR showed a decreasing trend for the viral RNA amount along the increased CE length, consistent with the symptom development and the immunoblot results (Figure 5C). The retention of the inserted GFP cassette was tested over time by RT-PCR, the expected size of the GFP expression cassette was detected in all plants tested, but a few plants showed some very faint bands of lower size at 5 wpi, indicating partial deletion of the inserted cassette (Figure 5D). Taken together, these results showed that all the different LIYV CP CE constructs tested were capable of foreign gene expression, however in our experiments the 150 nt was the shortest CE sequence that showed both high GFP expression and LIYV CP accumulation and was most stable for at least 5 weeks. Therefore, the LIYV-L2C_GFP_ construct comprising the 150 nt LIYV CP CE sequence was used for the remaining experiments.

### 3.5. Whitefly Transmissibility of LIYV-L2C_GFP_

**Figure 6:**
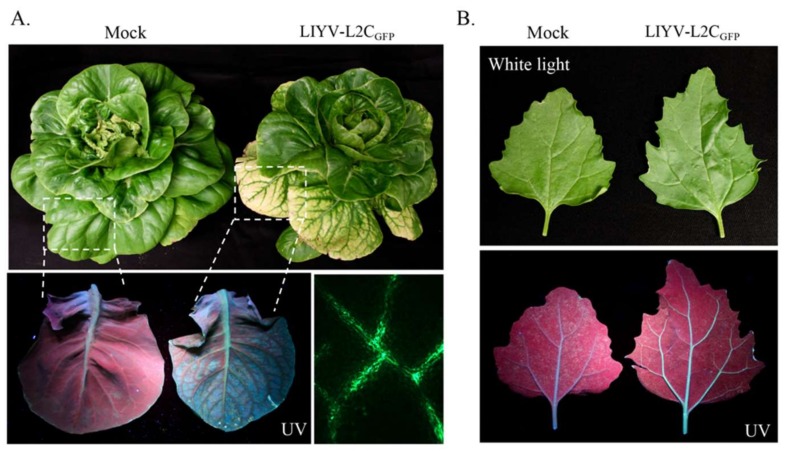
Systemic infection and GFP expression observed on LIYV-L2C_GFP_ infected *Lactuca sativa* L. and *Chenopodium murale* plants. Inoculation was performed with *Bemisia tabaci* that had fed on a sucrose diet containing LIYV-L2C_GFP_ particles purified from infected *Nicotiana benthamiana* plants. (**A**) Phenotypes of *L. sativa* L. plants inoculated with *B. tabaci* fed with buffer (Mock) and LIYV-L2C_GFP_ virions at 3 wpi. GFP fluorescence confined to the vascular tissues was visualized under UV light and fluorescence microscopy (lower panels). Dotted lines indicate enlarged areas. (**B**) Upper leaves of buffer (Mock) and LIYV-L2C_GFP_ inoculated *C. murale* plants photographed under white and UV light. GFP fluorescence was restricted in the veins.

Since the agroinoculation system of LIYV infectious clone is only practical for permissive *N. benthamiana* plants, and because LIYV has a wide plant host range, the ability of LIYV-L2C_GFP_ to be transmitted by its natural vector, the whitefly *B. tabaci*, to other host plants and to express inserted sequences was assessed. *B. tabaci* do not efficiently feed on *N. benthamiana* plants and thus cannot acquire LIYV directly from agroinoculated plants, therefore virions were purified from LIYV-L2C_GFP_-agroinoculated plants. Nonviruliferous *B. tabaci* were allowed to feed on a sucrose diet containing purified LIYV-L2C_GFP_ particles, and then transferred to six three-to-four leaf stage lettuce plants (*Lactuca sativa* L.) and six *Chenopodium murale* plants for virus inoculation [25]. All plants inoculated by viruliferous *B. tabaci* became infected and showed typical LIYV symptoms 3–4 weeks post inoculation (Figure 6). GFP expression was observed in systemically infected lettuce and *C. murale* plants by exposing plants to UV-light and by fluorescence microscopy (Figure 6). Like for *N. benthamiana* plants, fluorescence was most apparent in older leaves showing typical interveinal yellowing symptoms, and was restricted to veins.

### 3.6. Insertion of a Large Gene

**Figure 7:**
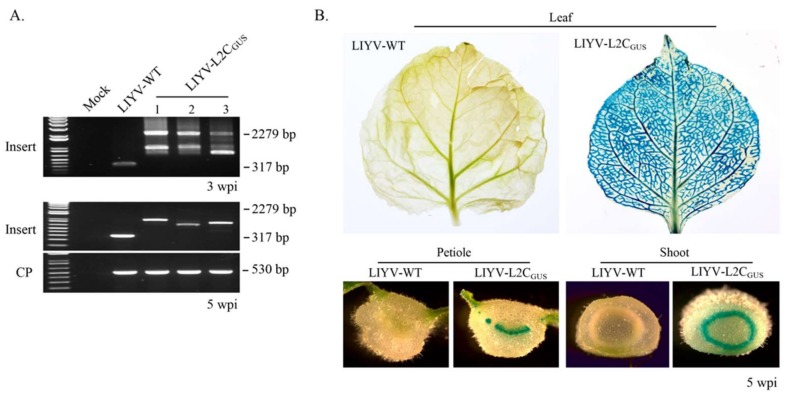
Viral infection and GUS activity in LIYV-L2C_GUS_ inoculated Hc-Pro *Nicotiana benthamiana* plants. (**A**) Detection of viral infection and insertion integrity by RT-PCR with total RNA extracted from upper non-inoculated leaves at 3 wpi and 5 wpi. Two primer sets were used to amplify the sequence of LIYV CP (CP, 530 bp) and the sequence flanking the GUS cassette (Insert, 2279 bp), LIYV-WT without the insert was amplified as a control (317 bp). The bands in between indicate partial deletions of the GUS cassette. (**B**) Systemic spread and distribution of GUS activity monitored in intact leaves and the cross-sections of petioles and shoots of LIYV-L2C_GUS_ infected plants at 5 wpi. Blue stain indicates GUS activity and was detected only in the vascular tissues. Tissues from LIYV-WT infected plants are applied as a negative control.

GFP is encoded by a relatively small ORF of only 720 nt. Because LIYV virions are rod-shaped filaments, there may not be such severe size restrictions as to the size of sequences that can be inserted into the LIYV genomic RNA2 and still be encapsidated. Therefore, to examine the expression of a larger gene in the LIYV-L2C_GFP_ vector, the 720 nt GFP ORF was replaced with the 1812 nt β-glucuronidase (GUS) ORF, referred to as LIYV-L2C_GUS_. This construct was tested on Hc-Pro *N. benthamiana* plants delivered by agroinoculation, LIYV-WT was used as a negative control. Systemic infection was successfully obtained with LIYV-L2C_GUS_, and similar to LIYV-L2C_GFP_, symptoms caused by LIYV-L2C_GUS_ were obvious at ca. 3 wpi, approximately one week later than on LIYV wildtype-infected plants, and symptoms were milder. To test the stability of LIYV-L2C_GUS_, we investigated expression cassette retention over time in *N. benthamiana* plants using RT-PCR with primers for the inserted cassette, primers amplifying the CP region were used as a control. Total RNA was extracted from the upper non-inoculated leaves of *N. benthamiana* plants infected by LIYV-WT and LIYV-L2C_GUS_ at 3 wpi and 5 wpi. A 2279 bp fragment comprising the complete GUS gene and several by-products of lower size, likely amplified from partially deleted genomes were detected from LIYV-L2C_GUS_ infected plants at 3 wpi, while 2 weeks later, only fragments of variant partial deletions were detectable by PCR, indicating the instability of the GUS coding sequence (Figure 7A). However, GUS activity was investigated in intact leaves, petioles and shoots of plants at 3 wpi and 5 wpi, and all showed strong GUS signals restricted in the vascular tissues, even at 5 wpi (Figure 7B).

### 3.7. VIGS in N. benthamiana Plants

**Figure 8:**
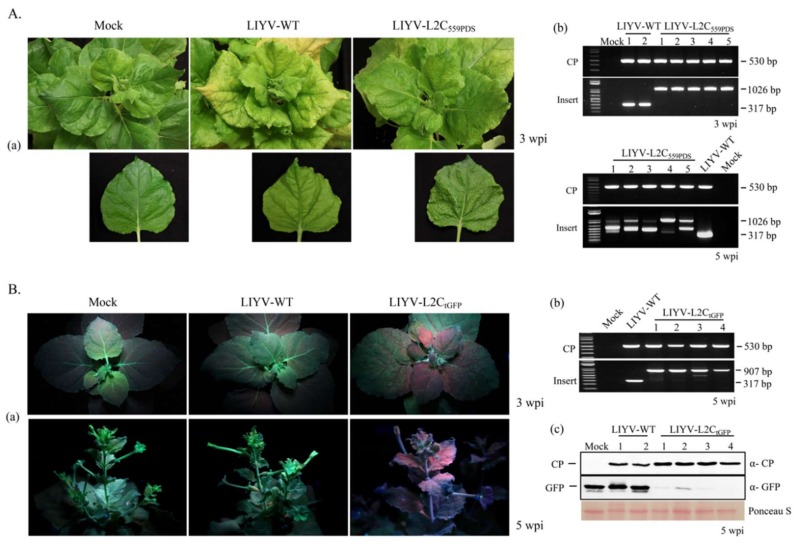
Virus-induced gene silencing in plants infected with LIYV vectors. (**A**) Silencing of the *phytoene desaturase* (PDS) gene with LIYV-L2C_tPDS_ in Hc-Pro transgenic *N. benthamiana* plants. (**a**) Phenotypes of LIYV-WT and LIYV-L2C_tPDS_ infected *N. benthamiana* plants photographed at 3 wpi; (**b**) Detection of viral infection and insertion integrity by RT-PCR with total RNA extracted from upper non-inoculated leaves at 3 wpi and 5 wpi. Two primer sets were used to amplify the sequence of LIYV CP (CP, 530 bp) and the truncated PDS cassette (Insert, 1026 bp), LIYV-WT without the insert was amplified as a control (317 bp). The bands in between at 5 wpi indicate partial deletions of the inserted cassette. (**B**) Silencing of GFP transgene with LIYV-L2C_tGFP_ in 16c transgenic *N. benthamiana* plants. (**a**) Silencing effects of LIYV-L2CtGFP observed under UV light at 3 wpi and 5 wpi. Plants inoculated with buffer (Mock) and LIYV-WT were negative controls; (**b**) Viral infection and insertion integrity were assessed by RT-PCR with total RNA extracted from upper non-inoculated leaves at 5 wpi. Two primer sets were used to amplify the sequence of LIYV CP (CP, 530 bp) and the truncated GFP cassette (Insert, 907 bp); (**c**) Immunoblot analysis of LIYV CP and GFP accumulation in upper non-inoculated leaves of LIYV-WT and LIYV-L2C_tGFP_ infected plants using LIYV CP and GFP specific antibodies. The Ponceau S stained Rubisco large subunit serves as a loading control.

In addition to recombinant protein expression, virus vectors can also be used for VIGS and reverse genetics. To assess the VIGS capabilities of LIYV vector, LIYV-VIGS vectors carrying truncated *phytoene desaturase* (PDS) and GFP genes were constructed, named as LIYV-L2C_tPDS_ and LIYV-L2C_tGFP_, targeting the endogenous PDS gene of *N. benthamiana* and GFP transgene of *N. benthamiana* line 16c respectively. We engineered a 559-nt PDS sequence and a 440-nt GFP sequence fragments replacing the GFP ORF of LIYV-L2C_GFP_ respectively under LIYV CP CE. A photo-bleaching phenotype in the vascular tissues of the newly emerged leaves was observed in LIYV-L2C_tPDS_ agroinoculated Hc-Pro *N. benthamiana* plants at ca. 3 wpi. RT-PCR-based analysis for retention of the inserted sequence showed good retention at 3 wpi, but instability of the insertion was observed at 5 wpi (Figure 8A).

The LIYV-L2C_tGFP_ construct was also tested on transgenic 16c *N. benthamiana* plants expressing GFP. The progress of GFP silencing was monitored using a long-wave UV lamp. Loss of fluorescence due to LIYV-VIGS was obvious by ca. 2 wpi, mostly along the veins of the agroinfiltrated plants. VIGS based silencing expanded into other areas gradually over time (Figure 8B), verifying the mobility of RNAi signal as described for *Arabidopsis* [42,43]. Systemic infection and retention of LIYV-L2C_tGFP_ cassette was checked by RT-PCR, the 440-nt truncated GFP fragment was mostly retained at 5 wpi, with some smaller amplicons suggesting some loss of insert (Figure 8B). Moreover, immunoblot analysis of total protein from the systemic leaves confirmed LIYV infection and significant downregulation of the GFP protein level in LIYV-L2C_tGFP_ infected plants compared to LIYV-WT and mock inoculated plants (Figure 8B).

## 4. Discussion

The family *Closteroviridae* includes four genera: *Closterovirus*, *Crinivirus*, *Ampelovirus* and *Velarivirus.* All viruses in this family have filamentous rod-shaped virions and posseses the largest genomes of plant-infecting positive-sense single-stranded RNA viruses. Their large, complex genomes and because they infect several economically important crop plants such as citrus, grapevines and cucurbits have drawn interest to develop closterovirus-based vectors for both fundamental and practical applications. So far, BYV, *Citrus tristeza virus* (CTV), and *Grapevine leafroll-associated virus* 2 (GLRaV-2), all members of the genus *Closterovirus,* have been developed into efficient gene expression vectors capable of infecting their host plants systemically and exhibiting strong potential for application in functional genomics and possibly pest and/or pathogen control [44,45,46]. However, until the present work no crinivirus-derived vector has yet been available. Criniviruses have genomes that are bipartite in contrast to the monopartite genome of viruses in other genera of the family *Closteroviridae* [47]. The genomic RNAs are separately encapsidated into virions approximately half the length of the virions seen for monopartite viruses of the genus *Closterovirus*. Could criniviruses have advantages over closterovirus-based vectors? Can sequences be inserted into both of the genomic RNAs? Can the recombinant viruses retain insect vector transmissibility and induce desired effects in different host plant species? In this study we addressed some of these questions. We developed a crinivirus-derived vector using LIYV, which is capable of systemic infection for efficient protein expression and VIGS in different plant species. This can be delivered by agroinoculation to *N. benthamiana* plants, and by whitefly transmission to other species that are recalcitrant to agroinoculation. This LIYV vector thus provides a valuable tool for studying plant-microbe interactions, functional genomics, and possibly even approaches for pest and/or pathogen control.

The first successfully developed closterovirus vector, BYV, was constructed through the “add-a-gene” strategy, that involved the splicing of an autonomous cassette controlled by an additional sgRNA CE, and this has become a prototype for other closterovirus-based vectors [45,46,48]. Based on the properties of closteroviruses that the sgRNAs for ORFs closer to the 3′ terminus of the genome tend to accumulate to higher levels [49], we inserted the GFP reporter cassette separately in between the last ORF and 3′-NTR of clones for both LIYV genomic RNAs. However, bright fluorescence was only observed with the RNA2 construct and fluorescence was limited to phloem tissues and was strongest in leaves with viral symptom development. LIYV accumulation, as determined by immunoblot analysis for the CP, indicated lower amounts of CP compared to wildtype virus, likely because of the increased genome size and the additional promoter [50]. The infectivity of the RNA1 construct was abolished when the GFP cassette was inserted near the RNA 1 3′ region. Considering that LIYV RNA1 encodes proteins critical for virus replication, the addition of the exogenous gene may have disrupted some of its necessary functions.

Another common strategy to express foreign genes from a viral vector is to replace part of the virus genome that encodes a non-essential protein. Based on our knowledge of LIYV, the appropriate functions of all genes are essential for efficient systemic infection, but mutations of either P5 or P9 genes only results in reduced virus accumulation without disrupting the systemic infectivity of LIYV in *N. benthamiana* plants [22]. The P5 ORF was therefore replaced by the GFP ORF to test the possibility of constructing a LIYV vector through the “substitution” strategy. Not surprisingly, the substituted LIYV vector was capable of systemic GFP expression in *N. benthamiana* plants, but at a much-reduced efficiency compared to the “add-a-gene” construct. This might be because P5 is the only LIYV-encoded protein that is translated from the full-length RNA2, and full-length RNA2 also must become encapsidated for progeny virion accumulation, as well as the lack of possible P5 functions on viral infection.

Of the closterovirus expression vectors examined previously, the choice of an appropriate CE is important and can affect the efficiency of foreign gene expression. The CP sgRNA CE directs gene expression and to a high level and is often employed to drive foreign gene expression [50]. Empirically, addition of a duplicated sequence in viral genomes increases its risk of being eliminated via homologous recombination, i.e., reduces vector stability [51]. Therefore, a heterologous CP sgRNA CE from a related virus has been commonly introduced for constructing closterovirus-derived vectors [40,45,46,48]. However, CTV vectors exhibited high tolerance to the duplicated homologous sequence, the extra gene controlled by a homologous or heterologous CP sgRNA CE showed no obvious effect on the expression level and stability of CTV construct [46]. Similar to CTV vectors, the GFP expression cassette driven by a duplicated LIYV CE showed high protein expression and genome stability. Surprisingly, when we replaced LIYV CP sgRNA CE with that of another crinivirus, CYSDV, a similar virus accumulation level and retention of the intact expression cassette were detected systemically, but no GFP expression was obtained. The activity of the heterologous CYSDV CE might have been inhibited without its natural genetic background, thus might represent viral specificity in the generation of the subgenomic RNAs. Moreover, when BYV CP sgRNA CE was used, no sign of LIYV infection was detected in the agroinoculated plants. It is yet unclear how the added BYV CE abolished LIYV infectivity, but may indicate low tolerance of criniviruses to foreign functional elements.

The effect of different sizes of the LIYV CE was also examined for optimal expression, since the LIYV sgRNA CE has not been characterized and an arbitrary sequence was selected for the first trials. Unlike that observed with the heterologous BYV sgRNA CE used in CTV vectors, in which no clear relationship was found between the size of the CE and gene expression [46], the longer CE used in the LIYV vector was shown to be preferable for higher GFP expression, but lower virus accumulation. Furthermore, the capacity of LIYV vector to express large inserts was tested by expressing the ~2 kb GUS gene. Instability of the insertion was noticed over time; however, good GUS activity was continuously produced at the very late stage of the infected *N. benthamiana* plants. This shows the ability of LIYV to harbor and express a large gene, but it is not as stable as some other closterovirus vectors such as GLRaV-2 and CTV that have demonstrated the ability to accommodate a gene of similar size stably over a year in their host plants [36,45].

The ability of LIYV vectors for VIGS was validated by silencing the endogenous gene PDS in Hc-Pro transgenic *N. benthamiana* plants, and the transgene GFP in *N. benthamiana* line 16c plants. The photo-bleaching phenotype was observed continuously in the veins of Hc-Pro *N. benthamiana* plants infected with LIYV vector harboring a truncated PDS fragment. It is interesting to note the efficiency of the LIYV VIGS vector induced silencing in the Hc-Pro *N. benthamiana* plants as these plants constitutively express Hc-Pro, a potent suppressor of RNAi activity. The Hc-Pro silencing suppressor has been reported previously to suppress VIGS [52], but LIYV induced VIGS was unaffected in the vascular tissues. By contrast, a strong silencing effect was visualized with the GFP construct starting from the vascular system then expanding to other areas, which is consistent with the cell-to-cell and systemic transport of RNAi signal described before [53].

The LIYV-based vectors developed here have exhibited a dual capacity for recombinant gene expression in the phloem, and for systemic silencing targeting endogenous host genes and transgenes in *N. benthamiana* plants. When combined with the ability of being transmitted by whitefly vectors and the wide host range of LIYV, LIYV-based vectors can be a valuable research tool, especially for research relevant to the vascular system. It also provides a model that could be applicable to other economically important criniviruses and their specific host plants.
